# Supplementary material for: Assessment of Common Risk Factors of Non-Communicable Diseases (NCDs) and Periodontal Disease in Indian Adults: An Analytical Cross-Sectional Study
Source: Methods Protoc. 2022 Mar 3;5(2):22. doi: 10.3390/mps5020022 (PMC8938845; doi:10.3390/mps5020022)
Supplement: Supplementary file 1 [file mps-05-00022-s001.zip › mps-1581311 supplementary Section S1-Data collection forms.pdf]

Section S1-Data collection forms

CRF-NCD/PD FORM 5

Data collection form for eligibility for inclusion

Name:

Case Number:

CRF-NCD/PD FORM 5

Name: O.P.No.:

Age: Date:

Sex: Contact No.:

Occupation: Address:

Medical/Dental history

Non-communicable Disease	Present/Absent
Diabetes Mellitus	
Hypertension	
Coronary heart disease (heart attack)	
Cerebrovascular disease (stroke)	
Peripheral vascular disease	
Periodontal disease	
Other conditions	

Eligible for inclusion in the study

Yes	No

<u>If eligible for inclusion, assign Patient ID Number:</u>

CRF-NCD/PD FORM 6

Data collection forms for recording of anthropometric data

Patient ID:

Serial No:

Age:

Anthropometric data

[Four anthropometric measures are commonly registered in the health care: weight, height, waist circumference (waist), and hip circumference (hip).]

Parameter	
Age (Years)	
Weight (Kg)	
Height (Cm)	
Body Mass Index (BMI) (Kg/m2)	
Waist circumference (cm)	
Hip circumference (cm)	
Waste Hip Ratio (WHR)	

CRF-NCD/PD FORM 7

Diagnostic criteria for Diabetes mellitus

Patient ID:

Serial No:

Age:

Diabetes Mellitus

Diabetes mellitus type 2 is diagnosed if a patient has any two of the following findings:

- Have symptoms of diabetes (increased thirst, increased urination, and unexplained weight loss) and a blood sugar level equal to or greater than 200 milligrams per deciliter (mg/dL) in random plasma glucose test or random blood sugar (RBS) test
- Have a fasting blood sugar (FBS) level that is equal to or greater than 126 mg/dL. A fasting blood sugar test (fasting plasma glucose) is done after not eating or drinking anything but water for 8 hours.
- Have a 2-hour oral glucose tolerance test (OGTT) result that is equal to or greater than 200 mg/dL.
- Have a hemoglobin A1c that is 6.5% or higher. This test is most reliable for adults.

Symptoms of diabetes	Present/ Absent [cancel the incorrect answer]
RBS levels	
FBS levels	
OGTT levels	
HbA1c	

CRF-NCD/PD FORM 8

Diagnostic criteria for Hypertension

Patient ID:

Serial No:

Age:

Hypertension

Hypertension (high blood pressure) diagnosed by the following findings:

**Blood Pressure**	**SBP**	**DBP**
**Classification**	**mmHg**	**mmHg**
Normal	<120	<80
Prehypertension	120–139	80–89
Stage 1 Hypertension	140–159	90–99
Stage 2 Hypertension	≥160	≥100

Stages of hypertension	Please mark ‘x’ against the appropriate row
Normal blood pressure	
Pre hypertension	
Stage 1 hypertension	
Stage 2 hypertension	

CRF-NCD/PD FORM 9

Diagnosis of Coronary Heart Disease (CHD)/Myocardial Infarction (MI)

Patient ID:

Serial No:

Age:

Coronary Heart Disease (CHD)/Myocardial Infarction (MI)

Coronary heart disease (heart attack) diagnosed by abnormalities detected in any of the following tests:

- Electrocardiogram (ECG).
- Echocardiogram.
- Exercise stress test.
- Nuclear stress test.
- Cardiac catheterization and angiogram.
- Cardiac CT scan.
- A CT coronary angiogram,

CRF-NCD/PD FORM 9

Diagnosis of CHD	Please mark 'x' against the appropriate row and write the interpretation of the investigation
➢ Electrocardiogram (ECG).	
➢ Echocardiogram.	
➢ Exercise stress test.	
➢ Nuclear stress test.	
➢ Cardiac catheterization and angiogram.	
➢ Cardiac CT scan.	
➢ A CT coronary angiogram	

CRF-NCD/PD FORM 10

Diagnosis of Cerebrovascular disease(Stroke)

Patient ID:

Serial No:

Age:

Cerebrovascular disease(Stroke)

Cerebrovascular disease (stroke) diagnosed by abnormalities detected in any of the following tests:

- A physical exam.
- Blood tests
- Computerized tomography (CT) scan
- Magnetic resonance imaging (MRI)
- Carotid ultrasound
- Cerebral angiogram
- Echocardiogram.

CRF-NCD/PD FORM 10

Diagnosis of Cerebrovascular disease	Please mark 'x' against the appropriate row and write the interpretation of the investigation
➢ A physical exam/stroke scale	
➢ Blood tests	
➢ Computerized tomography (CT) scan	
➢ Magnetic resonance imaging (MRI)	
➢ Carotid ultrasound	
➢ Cerebral angiogram	
➢ Echocardiogram.	

CRF-NCD/PD FORM 11

Diagnosis of Peripheral vascular disease

Patient ID:

Serial No:

Age:

Peripheral vascular disease

Peripheral vascular disease diagnosed by abnormalities detected in any of the following tests:

- Physical exam
- Ankle-brachial index (ABI)
- Ultrasound
- Angiography
- Blood tests

Diagnosis of Peripheral vascular disease	Please mark 'x' against the appropriate row and write the interpretation of the investigation
➢ Physical exam	
➢ Ankle-brachial index (ABI)	
➢ Ultrasound	
➢ Angiography	
➢ Blood tests	

Diagnosis of Periodontal disease- CRF-NCD/PD-FORM 12

Patient ID:

Serial No:

Age:

Periodontal disease

Periodontal disease: Inflammatory process affecting the supporting structures of the teeth resulting in pocket formation, recession, or both.

Diagnosed with a Periodontal Screening and Recording(PSR) score of 3 in at least one sextant.

a. Oral Hygiene Index-Simplified

16	11	26
46	31	36

Debris Index (DI)

16	11	26
46	31	36

Calculus Index(CI)

OHI-S=DI +CI

b. Plaque Index (Sillness and Loe)

16	11	26
46	41	36

Buccal

16	11	26
46	41	36

Lingual

CRF-NCD/PD FORM 12

c. Periodontal Screening and Recording

CRF-NCD/PD FORM 13

Laboratory investigations

Patient ID:

Serial No:

Age:

Laboratory Investigations:

Blood Analysis:

RBS

HbA1c

FBS

OGTT

Lipid profile

Total Cholesterol

HDL

LDL

Triglycerides

CRF-NCD/PD FORM 14

Diagnosis and treatment

Patient ID:

Serial No:

Age:

Diagnosis

Treatment

CRF-NCD/PD FORM 15

Risk factor assessment Form-Part 1-Demographic data

Patient ID:

Serial No:

Age:

Gender

Address

Occupation

Religion

Access to care: Available/ Not available

Familial pattern

Disease	Mother	Father	Siblings	Other blood relatives
Coronary Heart disease/Myocardial Infarction (MI)				
Cerebrovascular disease (Stroke)				
Hypertension				
Diabetes Mellitus-Type 2(DM2)				
Peripheral Vascular disease				
Periodontal disease(PD)				

CRF-NCD/PD FORM 15

Family size

No. of Adults	No. of children	Total

Insurance: Yes/No

CRF-NCD/PD FORM 15.1

Risk factor assessment Form-Part 1-Demographic Data-Socioeconomic status

Patient ID:

Serial No:

Age:

Socioeconomic status (Modified Kuppuswamy Criteria

Level of education	1(Illiterate)	2 (Primary school certificate)	3(Middle school certificate)	4 (High school certificate)	5 (Intermediate of post high school diploma)	6 (Graduate of PG)	7(Professional degree)
Occupation of head of family	1(unemployed)	2 (unskilled worker)	3 (semi-skilled worker)	4 (skilled worker)	5 (clerical, shop owner, farm)	6 (Semi professional)	10 (professional-white collar)
Monthly income of family	1(≤2,640)	2 (2,641-7886)	3 (7887-13,160)	4 (13,161-19,758)	6 (19,759-26-354)	10 (26,355-52,733)	12 (≥52,734)
Socioeconomic class	I (26-29)	II (16-25)	III (11-15)	IV (5-10)	V(1-4)		

CRF-NCD/PD FORM 16

Risk factor assessment Form-Part 2- Obesity assessment form

Patient ID:

Serial No:

Age:

Body Mass Index(BMI)= Weight in Kilograms/square of height in meters(Kg/m^2)

Underweight(<18.5)	Normal(18.5-24.9)	Overweight(25-29)	Obese(30-40)	Morbid obesity(>40)

CRF-NCD/PD FORM 17

Risk factor assessment Form-Part 2- Tobacco usage assessment form

Patient ID:

Serial No:

Age:

Tobacco Usage: Yes/No

If yes:

	Duration	frequency	Quantity
Smokeless			
Smoking			
E-cigarette			
Other forms of tobacco			

CRF-NCD/PD FORM 18

Risk factor assessment Form-Part 2- Physical activity measurement form

Patient ID:

Serial No:

Age:

Physical activity measurement tool

High activity- Vigorous activity 3 times a week or more	Medium activity- vigorous activity 1-2 times per week	Low activity- moderate exercise 3 or more times per week with no regular weekly vigorous exercise	Sedentary-Moderate exercise less than 3 times per week with no regular vigorous exercise

Risk factor assessment Form-Part 2- Alcohol Consumption assessment form [AUDIT score]

Patient ID:

Serial No:

Age:

Question	0	1	2	3	4
How often do you have a drink containing alcohol?	Never	Monthly or less	2-4 times a month	2-3 times a week	4 or more times a week
How many drinks containing alcohol did you have on a typical day when you were drinking in the past year?	1 or 2	3 or 4	5 or 6	7,8 or 9	10 or more
How often did you have 6 or more drinks on one occasion in the past year?	Never	Less than monthly	Monthly	Weekly	Daily or almost daily
How often during the last year have you found that you were not able to stop drinking once you had started?	Never	Less than monthly	Monthly	Weekly	Daily or almost daily
How often during the last year have you failed to do what was normally expected from you because of drinking?	Never	Less than monthly	Monthly	Weekly	Daily or almost daily
How often during the last year have you needed a first drink in the morning to get yourself going after a heavy drinking session?	Never	Less than monthly	Monthly	Weekly	Daily or almost daily
How often during the last year have you had a feeling of guilt or remorse after drinking?	Never	Less than monthly	Monthly	Weekly	Daily or almost daily
How often during the last year have you been unable to remember what happened the night before because you had been drinking?	Never	Less than monthly	Monthly	Weekly	Daily or almost daily
Have you or someone else been injured as a result of your drinking?	No		Yes,but not in the last year		Yes,during the last year
Has a relative or friend or doctor or other health care worker been concerned about your drinking or suggested you cut down?	No		Yes,but not in the last year		Yes,during the last year
AUDIT SCORE	0-7	8-15	16-19	20-40	
RISK LEVEL	ZONE I	ZONE II	ZONE III	ZONE IV	

CRF-NCD/PD FORM 20

Risk factor assessment Form-Part 2- Food Frequency Questionnaire

Patient ID:

Serial No:

Age:

Over the past month, on a typical day (excluding fasting, festivals etc.), how often have you eaten [specific food item] and what was the average portion size that you consumed [prompting from models and standard portion sizes]?

No	Item	Unit	Amount eaten in a day
1	Tea with milk	Glass	
2	Coffee with milk	Glass	
3	Lime water with sugar	Glass	
4	Butter Milk	Glass	
5	Baked/fried wheat/grain bread	Number/Bowl	
6	Rice preparations	Number/Bowl	
7	Pulse preparations	Bowl	
8	Leafy vegetable preparations	Bowl	
9	Roots and tubers preparations	Bowl/Number	
10	Other vegetable preparations	Bowl	
11	Vegetables eaten in raw form	Bowl/Number	
12	Non-Veg. food preparations	Bowl/Number	
13	Chutnies	Spoon	
14	Pickles	Spoon	
15	Milk and milk products	Glass/bowl/spoon	

CRF-NCD/PD FORM 20

16	Miscellaneous	Spoon, Number, Bowl	
17	Fried snacks (all types)	Number, Bowl	
18	Fruits	Number	
19	Alcohol consumption	Millilitres	
20	Water	Glass	

CRF-NCD/PD FORM 21

Risk factor assessment Form-Part 2- Standard stress assessment questionnaire

Patient ID:

Serial No:

Age:

Please answer the following questions based on your experience within the **last month**.	Not at All	Little Bit	Somewhat Much	Quite a Bit	Very
1. How stressful would you say your life is?	1	2	3	4	5
2. Dealing with daily stresses is negatively affecting my daily tasks.	1	2	3	4	5
3. I have a high intake of sugar and/or processed foods.	1	2	3	4	5
4. I feel worn down and/or burnt out.	1	2	3	4	5
5. I need caffeine or other energy drinks in the morning or afternoon to give me energy.	1	2	3	4	5
6. I seem to have lower than usual energy during the day.	1	2	3	4	5
7. I experience body aches and pains.	1	2	3	4	5
8. I have periods of low moods.	1	2	3	4	5
9. I feel more irritable.	1	2	3	4	5
10. My weight and metabolism have changed.	1	2	3	4	5
11. I can't seem to focus or concentrate.	1	2	3	4	5
12. I have feelings of anxiousness.	1	2	3	4	5
13. I feel totally exhausted most of the day and only have a few productive hours.	1	2	3	4	5
14. I find myself pushing through fatigue to get things done.	1	2	3	4	5
15. I seem to be sleeping a lot but never feel quite rested. I wake up feeling tired.	1	2	3	4	5
16. I have difficulty getting to sleep and/or wake up in the middle of the night.	1	2	3	4	5
17. I experience strong cravings for sweet or salty foods.	1	2	3	4	5
18. I feel overwhelmed with daily tasks and all that is on my plate.	1	2	3	4	5
19. I have a low sex drive.	1	2	3	4	5
20. I am unable to enjoy socializing with family and/or friends.	1	2	3	4	5

Add up your total score and mark where you fall on the stress scale below. Total: ________________

Low Stress			High Stress	
20	40	60	80	100

CRF-NCD/PD FORM 21

Stress is fairly well managed in your life. It maybe important to support your body to continue its healthy response	Your body's response to stress may be getting in the way of normal activities quite frequently, leaving you feeling depleted. Consult your health care professional for an individualized program to achieve your health goals	You may have experienced prolonged stress, andyour body's stress response can no longer adaptor successfully cope. Consult your health care professional for targeted support and strategies for improvement

CRF-NCD/PD FORM 22

Risk factor assessment Form-Part 2- Oral Hygiene and Periodontal Status Assessment Form

Patient ID:

Serial No:

Age:

a. Oral Hygiene Index-Simplified

16	11	26
46	31	36

Debris Index (DI)

16	11	26
46	31	36

Calculus Index(CI)

OHI-S=DI +CI

b. Plaque Index (Sillness and Loe)

16	11	26
46	41	36

Buccal

16	11	26
46	41	36

Lingual

Periodontal Screening and Recording

